# 3D hand pose and mesh estimation via a generic Topology-aware Transformer model

**DOI:** 10.3389/fnbot.2024.1395652

**Published:** 2024-05-03

**Authors:** Shaoqi Yu, Yintong Wang, Lili Chen, Xiaolin Zhang, Jiamao Li

**Affiliations:** ^1^Shanghai Institute of Microsystem and Information Technology, Chinese Academy of Sciences, Shanghai, China; ^2^University of Chinese Academy of Sciences, Beijing, China; ^3^ShanghaiTech University, Shanghai, China

**Keywords:** 3D hand pose estimation, HandGCNFormer, 3D hand mesh estimation, Graphformer, Transformer, GCN

## Abstract

In Human-Robot Interaction (HRI), accurate 3D hand pose and mesh estimation hold critical importance. However, inferring reasonable and accurate poses in severe self-occlusion and high self-similarity remains an inherent challenge. In order to alleviate the ambiguity caused by invisible and similar joints during HRI, we propose a new Topology-aware Transformer network named HandGCNFormer with depth image as input, incorporating prior knowledge of hand kinematic topology into the network while modeling long-range contextual information. Specifically, we propose a novel Graphformer decoder with an additional Node-offset Graph Convolutional layer (NoffGConv). The Graphformer decoder optimizes the synergy between the Transformer and GCN, capturing long-range dependencies and local topological connections between joints. On top of that, we replace the standard MLP prediction head with a novel Topology-aware head to better exploit local topological constraints for more reasonable and accurate poses. Our method achieves state-of-the-art 3D hand pose estimation performance on four challenging datasets, including Hands2017, NYU, ICVL, and MSRA. To further demonstrate the effectiveness and scalability of our proposed Graphformer Decoder and Topology aware head, we extend our framework to HandGCNFormer-Mesh for the 3D hand mesh estimation task. The extended framework efficiently integrates a shape regressor with the original Graphformer Decoder and Topology aware head, producing Mano parameters. The results on the HO-3D dataset, which contains various and challenging occlusions, show that our HandGCNFormer-Mesh achieves competitive results compared to previous state-of-the-art 3D hand mesh estimation methods.

## 1 Introduction

Robots can complete many repetitive and complex tasks for humans, and the development of computer vision technology enables robots to perform hand pose and mesh estimation. Accurate and robust 3D hand pose and mesh estimation is crucial in various Human-Robot Interaction (HRI) applications, including augmented reality, virtual reality, and third-person imitation learning.

**Figure 1:**
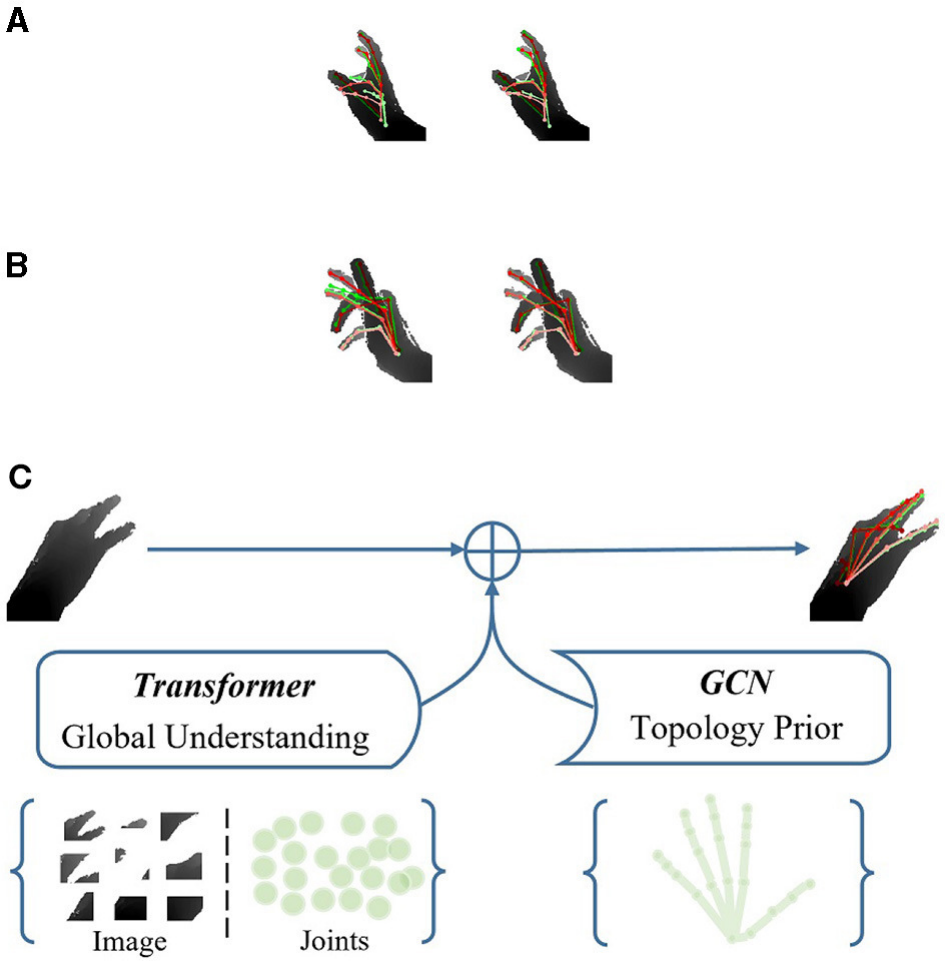
The illustration of the HandGCNFormer. **(A, B)** Respectively indicate qualitative comparison between AWR (left) and our HandGCNFormer (right) under self-occlusion and self-similarity. Red pose is the ground truth. Green pose represents predicted result. **(C)** Indicates the complementary feature representation of Transformer and GCN in HandGCNFormer.

Hand pose estimation seeks to ascertain the spatial coordinates of hand joints from either a single depth image or an RGB image. Depth-based methods have made impressive progress as commodity depth cameras become cheaper and more accurate (Guo et al., 2017b; Ren et al., 2019, 2021a; Xiong et al., 2019; Chen et al., 2020; Fang et al., 2020; Huang et al., 2020a,c). However, in situations where there is significant self-occlusion and high self-similarity in hand poses, it remains highly challenging, as illustrated in Figures 1A, B.

With the deep understanding of the scene and prior solid knowledge of hand kinematic structure, humans can accurately predict hand poses in complex scenarios, mitigating ambiguity caused by invisible and similar joints. Despite CNN-based hand pose estimation approaches (Ren et al., 2019; Xiong et al., 2019; Chen et al., 2020; Huang et al., 2020c) dominating the field, their inability to model long-range dependencies stems from operating on fixed-sized windows. To overcome this limitation, recent approaches (Huang et al., 2020a,b) leverage superior global modeling capability of the Transformer model to achieve better performance. However, they only implicitly extract the long-range dependencies behind the similarity of joint features while neglecting the natural kinematic constraints of hand topology.

The kinematic topology of the hand reveals the intrinsic connections between its joints. Previous studies (Zhao et al., 2019; Bai et al., 2021; Tunga et al., 2021) have demonstrated the capability of graph convolutional networks (GCNs) to represent such topological relationships. Recently, the pose-guided hierarchical graph convolution (PHG) method (Ren et al., 2021a) attempts to model the long-range dependencies between hand components by stacking multiple GCN layers. However, the cascaded GCNs can lead to error accumulation in long-term graph elements and worsen the problem of over-smoothing.

As depicted in Figure 1C, we believe that the global attention of the Transformer and the local topological awareness of GCNs construct feature representations that effectively combine and excel in modeling both short-range and long-range dependencies. To maximize the synergy between Transformer and GCN, we propose a novel Topological-aware Transformer network named **HandGCNFormer**. It integrates non-autoregressive Transformers for modeling contextual information from depth images and long-range dependencies between joints. Additionally, it employs Graph Convolutional Networks (GCNs) to naturally incorporate the prior knowledge of hand topology into our network and explicitly learn the relative relationships between locally connected joints.

Specifically, we propose a **Graphformer decoder**. Each decoder block contains a novel node-offset graph convolutional layer (NoffGConv) in the front, followed by standard components, including self-attention and cross-attention layers. Unlike vanilla GCN, NoffGConv decouples the node and offset feature mapping, enhancing the guidance of its location information in the feature aggregation process.

Another issue is that most Transformer-based methods utilize multiple-layer perception (MLP) heads composed of fully connected layers to independently predict the coordinates of hand joints, which overlooks the local connections between joints. Therefore, we introduce the **Topological-aware head** based on semantic graph convolutional layers (SemGConv; Zhao et al., 2019), which introduces topological information without increasing model complexity. SemGConv can obtain complex local spatial constraints between hand joints guided by hand topology through learnable adjacency matrices, thereby enabling the Topological-aware head to produce more reasonable and accurate poses.

Furthermore, 3D hand mesh estimation is also a current hot topic in research. Although extensive research has focused on 3D pose estimation of hands and objects using depth cameras (Moon et al., 2018; Li et al., 2020) or RGB-D sensors (Garcia-Hernando et al., 2018) in controlled environments within computer vision, recent research has shown promising results in pose estimation solely from a single RGB image. Our Graphformer decoder can generate shape Queries and Pose Queries, which can be processed by the proposed Topology-aware head and the MANO layer, respectively, and output the final 3D hand mesh. We adapted the original HandGCNFormer to the new HandGCNFormer-Mesh for 3D hand mesh estimation. Our HandGCNFormer-Mesh performs well in 3D hand mesh estimation when given a single RGB image as input, which demonstrates the effectiveness and scalability of our proposed Graphformer decoder and Topology-aware head in addressing not only 3D hand pose estimation from a single depth image, but also 3D hand mesh estimation from a single RGB image.

In a word, the main contributions of this paper are summarized as 3-fold:

- We propose a novel HandGCNFormer network for 3D hand pose estimation from a single depth image. Transformer and GCN layers are deeply integrated to model both global understanding of the scene and local topology connections of hand joints. We also adjust the proposed network and obtain HandGCNFormer-Mesh for 3D hand mesh estimation from a single RGB image, showing that the proposed structure can achieve good results not only when the input is a depth image but also an RGB image.
- We propose a novel NoffGConv layer to decouple the node feature mapping and the offset feature mapping. Experimental results demonstrate that proposed NoffGConv layer outperforms popular GCNs on 3D hand pose estimation tasks. On top of that, a Topology-aware head module is designed to adaptively establish the spatial topology constraints, which outperforms the standard MLP prediction head.
- Our method achieves excellent performance on five challenging datasets. In particular, it is superior to the top-performing approach by a margin of 3.2 with 7.6% fewer parameters for unseen subjects hand in Hands2017, revealing its excellent generalization ability. Moreover, HandGCNFormer-Mesh can also perform well on the 3D hand mesh estimation task on HO-3D by inputting a single RGB image.

Compared with the conference version (Wang et al., 2023), we mainly expand the following contents for the present work:

- We extend our framework as HandGCNFormer-Mesh for the task of 3D hand mesh estimation, to demonstrate the effectiveness and scalability of our proposed Graphformer Decoder and Topology Aware Head on not only 3D hand pose estimation task from depth input but also 3D hand mesh estimation with RGB input modality.
- We further explored the structural design of the framework, in which we integrate a Shape Regressor with original Graphformer Decoder and Topology aware head, producing Mano parameters to obtain the final 3D hand mesh.
- We also add more abundant implementation details and analyses based on the conference paper, including tensor feature dimension description and network implementation details.
- Extensive experiments have been conducted on challenging HO-3D dataset which contains various and severe occlusions under RGB scenarios. And the results demonstrate that our HandGCNFormer-Mesh achieves competitive results compared to previous state-of-the-art 3D hand mesh estimation methods.

## 2 Related work

### 2.1 3D hand pose estimation

Deep neural networks have shown remarkable performance in 3D hand pose estimation, with methods typically categorized as regression-based, detection-based, or hybrid approaches based on the nature of their model output. Regression-based models (Oberweger et al., 2015; Guo et al., 2017a,b; Oberweger and Lepetit, 2017; Xiong et al., 2019; Chen et al., 2020; Caramalau et al., 2021; Hampali et al., 2022; Madadi et al., 2022) directly learn the mapping from input images to output joint coordinates or angles. For instance, DeepPrior (Oberweger et al., 2015) and DeepPrior++ (Oberweger and Lepetit, 2017) utilize a bottleneck layer to learn pose priors and then regress poses using fully-connected layers. To enhance the utilization of fine-grained features, Pose-REN (Chen et al., 2020) adopts multilevel cascade regression to refine predictions iteratively. Meanwhile, other approaches (Guo et al., 2017a,b; Xiong et al., 2019; Madadi et al., 2022) utilize feature-level local ensembles. Despite their high performance, these methods often need more model complexity. Detection-based approaches (Moon et al., 2017, 2018; Ge et al., 2018b; Ren et al., 2019, 2021b) typically generate dense probability maps for each joint from input sources such as depth images, point sets, or voxel sets. For instance, DenseReg (Wan et al., 2018) employs an encoder-decoder module to produce 3D heatmaps and unit vector fields, preserving richer spatial context. However, because the post-processing step of extracting joint coordinates from heatmaps is similar across these methods, they often cannot be trained end-to-end. Subsequently, hybrid approaches (Sun et al., 2018; Huang et al., 2020c; Malik et al., 2020; Ren et al., 2021a) emerged, combining the strengths of both regression-based and detection-based methods. For example, AWR (Huang et al., 2020c) transforms 3D hand joint coordinates into a 3D heatmap and unit vector field, providing direct supervision for joint position. Nevertheless, pure CNN-based methods require adjustments to capture global context effectively, given their restricted receptive field. This limitation poses challenges in addressing severe self-occlusion and self-similarity issues, which are prevalent in 3D hand pose estimation tasks.

### 2.2 3D hand mesh estimation

After the release of hand-object interaction benchmark datasets such as HO-3D (Hampali et al., 2020), several studies have been conducted on these datasets (Hasson et al., 2019, 2020; Hampali et al., 2020). Hasson et al. (2019) introduced innovative loss functions incorporating physical constraints for interacting hands and objects. HOnnotate (Hampali et al., 2020) identified 2D joint positions and adjusted hand model parameters [such as MANO (Romero et al., 2022)] by minimizing their loss function. Hasson et al. (2020) utilized photometric consistency across sequential frames differently, and they inferred meshes for both hands and objects, generating a warping flow through rendering. Subsequently, they applied a pixel-level loss to ensure photometric consistency between a reference frame and the frame warped using the inferred flow. Most of the above methods focus on modeling the interaction between hands and objects while ignoring the modeling of global contextual information.

### 2.3 Transformer in computer vision

Recently, the Transformer architecture (Vaswani et al., 2017) has seen applications in various domains, including image classification (Dosovitskiy et al., 2020; Liu et al., 2021), object detection (Carion et al., 2020; Zhu et al., 2020), and pose estimation (Yang et al., 2020; Lin et al., 2021; Mao et al., 2021; Hampali et al., 2022; Li et al., 2022). Notably, PRTR (Li et al., 2021a) and TFPose (Mao et al., 2021) visualize the dynamic decoding process within the Transformer decoder, showcasing the Transformer's potential for human pose modeling. In a closely related study, Hand-Transformer (Huang et al., 2020a) employs a non-autoregressive Transformer decoding mechanism to concurrently localize each joint. Unlike the autoregressive method, the non-autoregressive decoding enables real-time processing by removing the constraint of sequence dependence. However, detecting joints independently disregards the inherent adjacency relationship among joints, resulting in subpar performance, particularly for invisible and similar joints.

### 2.4 Graph convolutional network

GCN has gained increasing popularity in skeleton-based action recognition (Yan et al., 2018; Mart́ınez-González et al., 2021; Tunga et al., 2021) and 2D-to-3D pose estimation tasks (Kong et al., 2020; Qiu et al., 2020), owing to its effectiveness in representing arbitrary topological data. SemGCN (Zhao et al., 2019) is introduced to capture intricate semantic relationships among neighboring joints of the human body. HOPE-Net (Doosti et al., 2020) proposes an adaptive Graph U-Net for inferring joint locations in 3D space from 2D keypoints. These approaches for 2D-to-3D lifting underscore the significance of topology information in mitigating depth ambiguity. PHG (Ren et al., 2021a) endeavors to establish long-range dependencies among hand joints by leveraging a cascaded GCN module, achieving state-of-the-art performance. However, the cascaded GCN module tends to introduce noisy information from extended neighbor nodes exponentially while constructing global relationships, resulting in over-smoothing of the model. In this study, we harness the Transformer architecture to model global contextual information directly, thereby circumventing the limitations imposed by receptive field constraints. Concurrently, we integrate GCN to capture the hand kinematic topology, significantly enhancing the representation of spatial structural features.

## 3 Methodology

**Figure 2:**
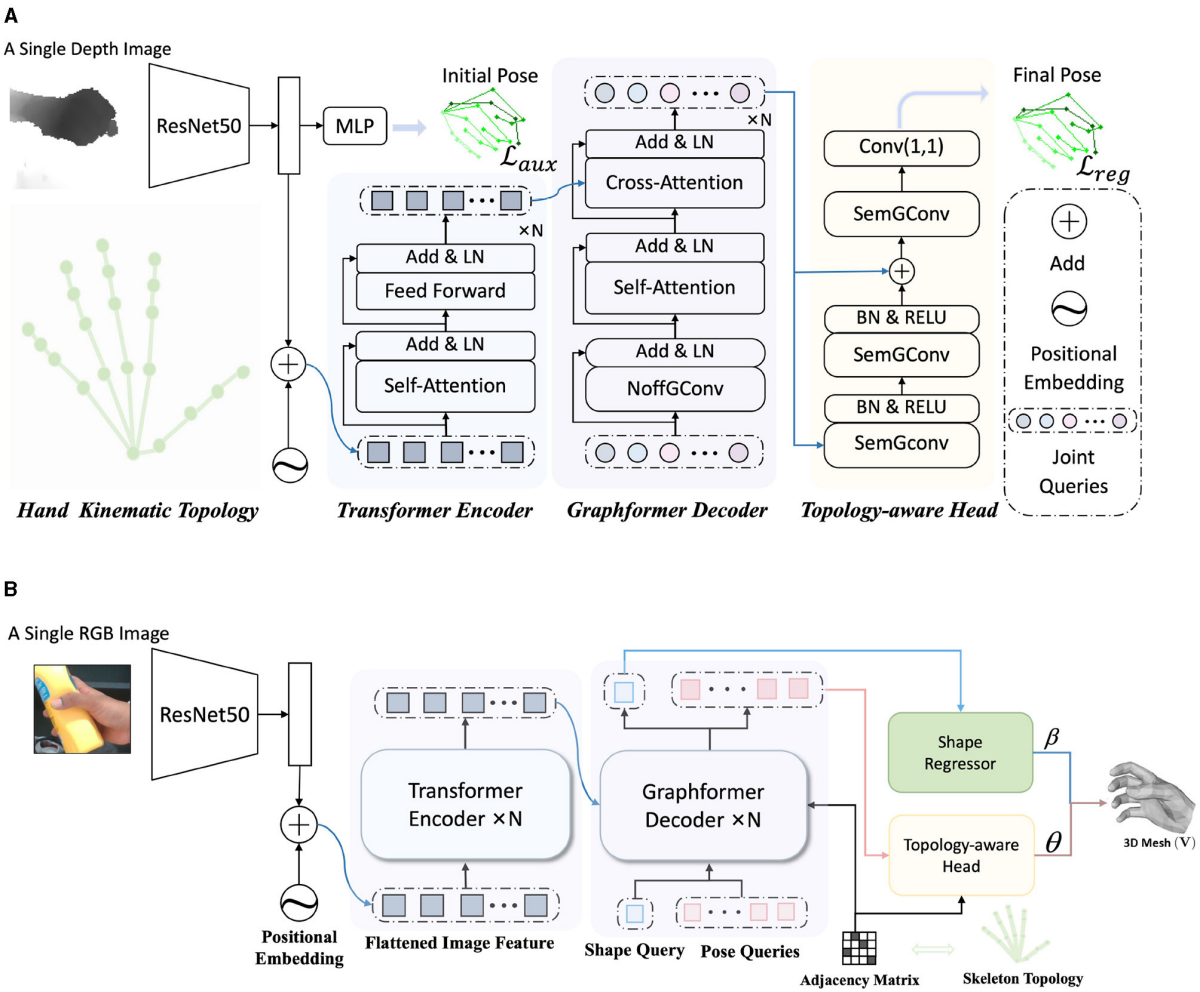
The overview of the HandGCNFormer. Our method introduces the prior knowledge of hand kinematic topology by NoffGConv and SemGConv layers, as well as models global understanding with self-attention mechanism, providing rich disambiguation evidence. ResNet and Transformer encoder form the image encoder module, capturing the global-local context of the image (Section 3.3). Graphformer decoder incorporates NoffGConv and attention modules capturing joint interaction globally without ignoring topological connections of joints (Section 3.4). Finally, the final pose is regressed by Topology-aware head while the final mesh is regressed by Topology-aware head and Shape Regressor. The Topology-aware head constructs effective topology constraints during regressing (Section 3.5). **(A)** An overall structure of the HandGCNFormer for 3D hand pose estimation. **(B)** An overall structure of the HandGCNFormer-Mesh for 3D hand mesh estimation.

Figure 2A depicts the schematic of our proposed HandGCNFormer. It takes a depth image as input and predicts a set of 3D joint coordinates. The framework comprises an image encoder, formed by a ResNet and a Transformer encoder, a Graphformer decoder, and a Topology-aware head. Furthermore, we explore the performance of HandGCNFormer on the 3D hand mesh estimation task, introducing HandGCNFormer-Mesh, as illustrated in Figure 2B.

### 3.1 Preliminary

#### 3.1.1 Transformer

It follows an encoder-decoder architecture and is designed completely depending on the self-attention mechanism. In the self-attention module, the dependencies of the input sequence are extracted by calculating the feature similarity. The input sequence **X**∈ℝ^*N*×*D*^ is mapped to queries **Q**∈ℝ^*N*×*D*^, keys **K**∈ℝ^*N*×*D*^, and values **V**∈ℝ^*N*×*D*^. *N* and *D* denote sequence length and dimensions, respectively. The scaled dot-product attention is computed as Equation 1:


(1) $$\begin{array}{l} \mathrm{Attention}\left(\text{Q},\text{K},\text{V}\right)=\mathrm{softmax}\left(\frac{\text{Q}{\text{K}}^{T}}{\sqrt{D}}\right)\text{V} \end{array}$$

Further, to jointly extract semantic information from different representation subspaces, Transformer introduces a multi-head self-attention (MHSA) module. The MHSA projects **Q**, **K**, and **V** into *h* subspaces as well as computing all attention heads in parallel. After that, the attention heads are concatenated and projected to the output of MHSA, which can be expressed as Equation 2:


(2) $$\mathrm{MHSA}(Q,K,V)=\mathrm{Concat}\left({\mathrm{Att}}_{1},\ldots {\mathrm{Att}}_{h}\right)W^{O}$$

### 3.2 Vanilla GCN

The graph G={V,E} consists of a series of nodes **V** and edges **E**. Assume that the input of the *l*-th layers of GCN is ${\text{X}}^{l}\in {\mathbb{R}}^{J^{*}D_{l}}$, *J* represents the number of nodes and *D*_*l*_ denotes input dimensions. To capture the relationship between node and associated neighboring nodes, GCN transforms the input feature **X**^*l*^ by a learnable matrix $\text{W}\in {\mathbb{R}}^{D_{l+1}\times D_{l}}$, and then aggregates the transformed information with a symmetric normalized matrix $\tilde{\text{A}}$. The convolution operation at the *l*-th layers is formulated as Equation 3:


(3) $$\begin{array}{l} {\text{X}}^{\left(l+1\right)}=\sigma \left({\text{W}}^{\left(l\right)}{\text{X}}^{\left(l\right)}\tilde{\text{A}}\right) \end{array}$$

where σ is the activation function and $\tilde{\text{A}}$ is computed by $\tilde{\text{A}}={\tilde{\text{D}}}^{-\frac{1}{2}}\left(\text{A}+\text{I}\right){\tilde{\text{D}}}^{-\frac{1}{2}}$. $\tilde{\text{D}}$ is a diagonal degree matrix. **A** is an adjacency matrix covering internal connections of G.

### 3.3 Image encoder

The image encoder is responsible for extracting both local and global features from the input depth image. Inspired by DETR (Carion et al., 2020), our image encoder comprises a ResNet (He et al., 2016) followed by a Transformer encoder. Given a cropped hand depth image **I**∈ℝ^*H*×*W*^, where *H* and *W* denote the image height and width respectively, a ResNet is employed to extract downsampled features $\text{F}\in {\mathbb{R}}^{\frac{H}{32}\times \frac{W}{32}\times 2048}$. These features are then channel-wise reduced using a 1 × 1 convolutional layer and spatially flattened to produce the sequence feature $\text{T}\in {\mathbb{R}}^{\frac{HW}{1024}\times 256}$, which is subsequently fed into the standard Transformer encoder. To preserve spatial positional information, sinusoidal positional embedding is incorporated into the input sequence. Finally, the context features of the input sequence are captured through a series of self-attentions and feed-forward networks (FFN).

### 3.4 Graphformer decoder

The conventional Transformer decoder comprises self-attention layers, cross-attention layers, and feed-forward networks, which lack awareness of the inherent connections among joints described by hand kinematic topology (as shown in the lower left part of Figure 2A). To address this limitation, we introduce a Graphformer decoder that emphasizes the integration of attention mechanisms and GCN techniques, leveraging both the long-range dependencies and local topology connections among joints. Specifically, we construct a graph G={V,E} consisting of nodes **V** and edges **E**. Each node in the graph represents a hand joint. We incorporate prior knowledge of hand kinematic topology into the model through the adjacency matrix of G. An edge between node *i* and *j* exists if the corresponding two joints are connected in the hand kinematic topology.

**Figure 3:**
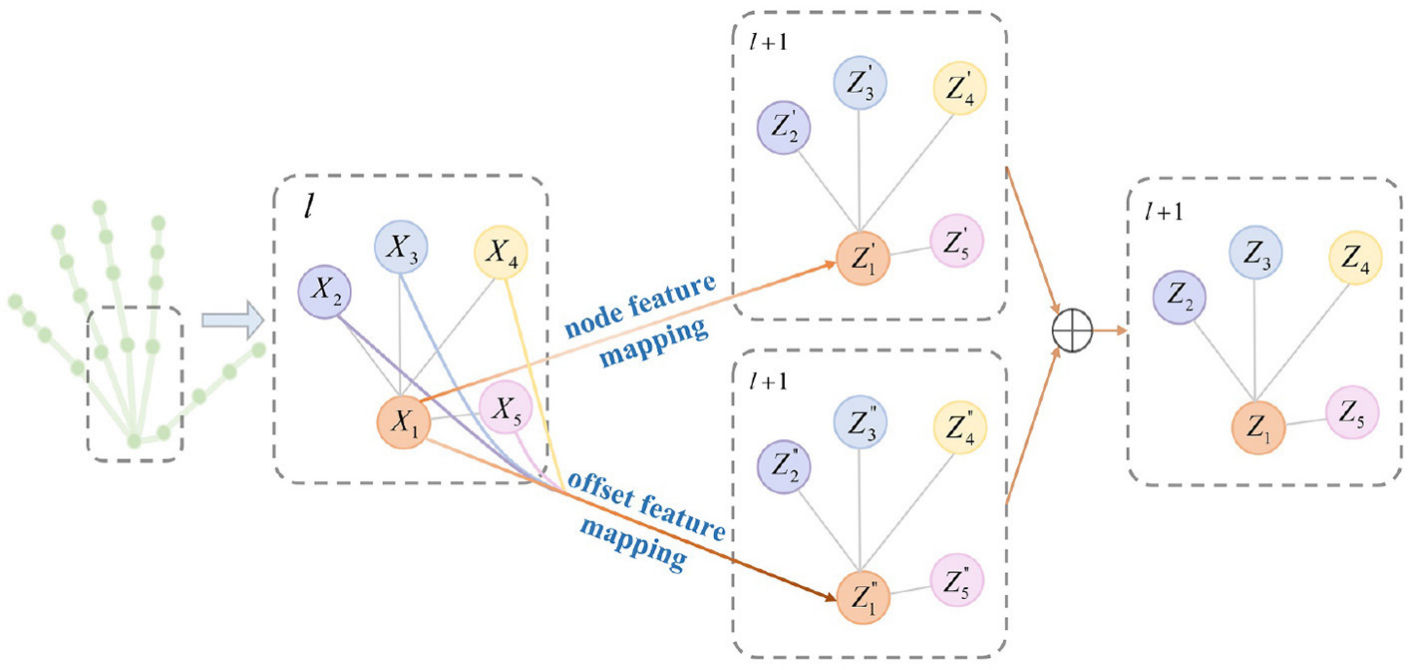
Illustration of NoffGConv. NoffGConv decouples the node feature mapping and the offset feature mapping during aggregating information. The gray lines indicate the connections between nodes, and the colored lines represent feature transfer.

In 3D hand pose estimation, node features carry abundant location information, while neighboring nodes contribute valuable features for estimating relative offsets, particularly beneficial for handling occluded or similar joints. Motivated by this insight, we introduce a node-offset graph convolutional layer (NoffGConv). Figure 3 illustrates how NoffGConv separates node and offset feature mapping: the former relies solely on node features, while the latter integrates refinement information from neighboring nodes and itself toward the central node. To better complement subsequent self-attention layers and expedite model convergence, NoffGConv employs a fixed adjacency matrix. Formally, considering the input of the *l*-th layer in NoffGConv as ${\text{X}}^{\left(l\right)}\in {\mathbb{R}}^{J\times D_{l}}$, where *J* denotes the number of nodes and *D*_*l*_ represents input dimensions, the NoffGConv at the *l*-th layer can be formulated as follows:


(4) $$\begin{array}{l} {\text{X}}^{\left(l+1\right)}=\sigma \left({\text{W}}_{1}{\text{X}}^{\left(l\right)}+{\text{W}}_{2}{\text{X}}^{\left(l\right)}\tilde{\text{A}}\right) \end{array}$$

where σ is the activation function and $\tilde{\text{A}}$ is the normalized adjacency matrix which is computed by $\tilde{\text{A}}={\tilde{\text{D}}}^{-\frac{1}{2}}\left(\text{A}+\text{I}\right){\tilde{\text{D}}}^{-\frac{1}{2}}$. $\tilde{\text{D}}$ is a diagonal degree matrix. **A** is an adjacency matrix covering internal connections of G. **I** is the identity matrix. With different weights **W**_1_ and **W**_2_, NoffGConv decouples the mapping of the node features and the offset features. Note that the vanilla GCN only has the second term in Equation (4), which assigns attention to the current node and its neighbors based on the degree matrix, weakening the guidance of its location information.

The Graphformer decoder consists of *N* decoder blocks, with each block comprising a NoffGConv layer followed by a standard self-attention layer and cross-attention layer. Our decoder takes learned joint queries as input, representing the positional embedding of joints. Given the one-to-one correspondence between joint queries and hand joints, the Hungarian matching (Carion et al., 2020) is unnecessary. Moreover, as NoffGConv already implements the nonlinear mapping of joint queries, we can eliminate the feed-forward network typically following the attention module.

### 3.5 Topology-aware head

In scenarios with heavy self-occlusion and self-similarity, the inherent spatial structure of hand joints plays a crucial role in accurately predicting hand pose. To address the limitations of existing MLP heads in capturing spatial structure cues, we propose a Topology-aware head incorporating GCN techniques. As mentioned earlier, GCN naturally leverages the prior knowledge of hand kinematic topology by aggregating information from nodes and their neighboring nodes under the guidance of topology. However, vanilla GCN assigns fixed attention to the connections between joints, overlooking the complex semantic relationships among neighboring nodes. Our Topology-aware head is configured with three semantic graph convolution layers (SemGConv) followed by a 1 × 1 convolutional projection layer to address this. Unlike vanilla GCN, SemGConv introduces a learned weighting matrix **M**∈ℝ^*J*×*J*^ to adaptively model the connection strength between joints, expressed as Equation 5:


(5) $$\begin{array}{l} {\text{X}}^{\left(l+1\right)}=\sigma \left(\text{W}{\text{X}}^{\left(l\right)}\rho_{i}\left(\text{M}⊙\left(\text{A}+\text{I}\right)\right)\right) \end{array}$$

where **W** represents a transformation matrix; ρ_*i*_ is the Softmax nonlinearity, which normalizes the weight of connections between a node *i* and its neighboring nodes j∈N(i); ⊙ denotes element-wise multiplication.

In line with prior research (Zhao et al., 2019), we employ residual connections to mitigate the issue of over-smoothing when stacking multiple SemGConv layers. Additionally, we concatenate the output embeddings from all Graphformer decoder layers and feed them collectively into our head module, promoting the network to implicitly extract semantic information across various decoder layers. Leveraging the advantageous properties of SemGConv, our regression head guides the pose to a more accurate space constrained by hand topology.

### 3.6 HandGCNFormer-Mesh

To further demonstrate the robustness and effectiveness of our proposed Topology aware head and Graphformer Decoder, we extend our framework as HandGCNFormer-Mesh for the task of 3D hand mesh estimation, as shown in the Figure 2B.

The input of the network is an RGB image, which is still processed through ResNet and directly input into the Transformer network. At the same time, we adjusted the design of the input learnable token of Graphformer Decoder, including Shape Query S, which is composed of one learnable token, and Pose Queries P, which is composed of multiple learnable tokens. After being processed by Graphformer Decoder, they will be input to Shape Regressor and Topology-aware head, respectively.

#### 3.6.1 Shape Regressor

We propose Shape Regressor in order to process Shape Query S. Shape Regressor consists of a 3-layer neural network, each comprising a Linear layer and a RELU activation function. The Shape Regressor and Topology-aware head will continue processing the output from the Graphformer Decoder.

Shape Regressor and Topology-aware head will output shape parameters β∈ℝ^10^ and pose parameters θ∈ℝ^48^, respectively. We multiplied the joint regression matrix to a 3D mesh in rest pose, applied the forward kinematics to get the final 3D hand joints coordinates, and obtained the final 3D hand mesh *V*∈ℝ^778 × 3^.

### 3.7 Overall loss function

#### 3.7.1 3D hand pose estimation task

The pose estimation task often yields a relatively sparse distribution of prediction results. Given that the Laplace distribution is more suitable for sparse data, the model is trained using a smooth L1 loss (Huang et al., 2020c) to minimize the error between the estimated and ground truth poses, considering both 2D and 3D poses. Let $y_{2D}\in {\mathbb{R}}^{J\times 2}$ and $y_{3D}\in {\mathbb{R}}^{J\times 3}$ denote the ground truth poses. The regression loss is formulated as follows in Equation 6:


(6) $${ℒ}_{reg}=\sum_{n=1}^{N}{\mathrm{smooth}}_{L1}\left({\hat{y}}_{2D}^{n},y_{2D}\right)+{\mathrm{smooth}}_{L1}\left({\hat{y}}_{3D}^{n},y_{3D}\right)$$

where ${ŷ}_{3D}^{n}$ denotes the predicted 3D pose from the output of the *n*-th decoder layer. ${ŷ}_{2D}^{n}$ is calculated by projecting ${ŷ}_{3D}^{n}$ with camera intrinsics.

Moreover, we employ a multilayer perceptron (MLP) on the ResNet backbone to predict an initial 3D pose consisting of three fully connected layers. An auxiliary loss is utilized to reinforce feature learning in the backbone and enhance overall performance, computed as follows in Equation 7:


(7) $$\begin{array}{l} L_{aux}={\mathrm{smooth}}_{L1}\left({\hat{p}}_{2D},y_{2D}\right)+{\mathrm{smooth}}_{L1}\left({\hat{p}}_{3D},y_{3D}\right) \end{array}$$

where ${\hat{p}}_{2D}$ and ${\hat{p}}_{3D}$ represent the 2D/3D coordinates corresponding to the initial pose, respectively.

Finally, the overall loss is the summation of the regression loss and auxiliary loss in Equation 8:


(8) $$\begin{array}{l} L_{overall}=L_{reg}+L_{aux} \end{array}$$

#### 3.7.2 3D hand mesh estimation task

To train the HandGCNFormer-Mesh, we minimize a loss function, defined as a combination of L2 distances between the predicted and ground truths as follows in Equations 9–13:


(9) $$\begin{array}{l} L_{\text{pose}}=||\theta -\hat{\theta }|{|}_{2} \end{array}$$

(10) $$\begin{array}{l} L_{\text{shape}}=||\beta -\hat{\beta }|{|}_{2} \end{array}$$

(11) $$\begin{array}{l} L_{\text{mesh}}=||V-\hat{V}|{|}_{2} \end{array}$$

(12) $$\begin{array}{l} L_{\text{joint}}=||J^{3D}-\hat{J^{3D}}|{|}_{2} \end{array}$$

(13) $$\begin{array}{l} L_{overall}=L_{pose}+L_{shape}+L_{mesh}+L_{joint} \end{array}$$

where *J*^3*D*^ denotes a 3D hand joint coordinates, obtained by multiplying a joint regression matrix to 3D hand mesh *V*, where the matrix is defined in MANO.

## 4 Experiments

### 4.1 Datasets

**Hands2017 dataset** (Yuan et al., 2017) contains 957 K training and 295 K testing images. Twenty-one hand joints are annotated.

**NYU dataset** (Tompson et al., 2014) contains 72 K training and 8.2 K testing images labeled with 36 joint locations. Following the common convention (Moon et al., 2018; Ren et al., 2021a), we pick a subset of 14 joints from the frontal view for evaluation.

**ICVL dataset** (Tang et al., 2014) contains 22 K training images and 1.6 K testing images. The training data is augmented to 330 K samples by leveraging in-plane rotation operations. The annotation of the pose contains 16 joints.

**MSRA dataset** (Sun et al., 2015) contains 76.5 K images with 17 gestures. The ground truth pose annotates 21 joints. We evaluate this dataset with the common leave-one-subject-out cross-validation strategy (Chen et al., 2020; Huang et al., 2020c).

**HO-3D dataset** (Hampali et al., 2020) is a hand-object interaction dataset that contains challenging occlusions. This dataset provides RGB images with MANO-based hand joints and meshes, and camera parameters. The dataset contains more than 65 sequences captured with 10 different subjects and 10 objects with both 3D pose annotations of hand and object. It has 66,034 and 11,524 hand-object interaction images from a third-person view for training and testing. The results on the test set can be evaluated via an online submission system.

### 4.2 Experimental settings

#### 4.2.1 Implementation details

We train our model end-to-end on a single NVIDIA 40GB A100 Tensor Core GPU. PyTorch framework is utilized for implementation, with the AdamW optimizer (Loshchilov and Hutter, 2017) and an initial learning rate of 0.0001. The batch size is set to 64. The training procedure spans 40 epochs, employing a multi-step learning rate schedule that decreases the learning rate by a factor of 0.1 at the 30th and 37th epochs, respectively. Our backbone architecture is ResNet-50, pretrained on ImageNet, with the remaining weights initialized using Xavier initialization (Glorot and Bengio, 2010). We employ eight heads for self-attention, and both the Transformer encoder and Graphformer decoder consist of four layers. During inference, we use the predictions from the final decoder layer as the final results. To determine the center coordinates of the hand region in 3D space, we adopt the localization network proposed in V2V-poseNet (Moon et al., 2018). Cropped images are resized to 256 × 256, and depth values are normalized to the range [−1, 1]. Data augmentation is performed in the world coordinate system, including random scaling, rotation, and translation. As per standard practice, a separate model is trained for each benchmark using its respective training set. In particular, for HandGCNFormer-Mesh, Shape Query contains one learnable token, while Pose Queries P contains 16 learnable tokens, and the token dimensions are 512.

### 4.3 Evaluation metrics

We assess our model using the same evaluation metrics as previous studies: (1) the mean 3D distance error and (2) the percentage of successful frames. The former calculates the average Euclidean distance error per joint between ground truth and predictions across the entire test set. The latter indicates the proportion of successful frames, where all joint errors are below a specified threshold relative to the total number of test frames.

### 4.4 Baseline

Our baseline follows the DETR (Carion et al., 2020) framework without the Hungarian matching algorithm. The input queries of decoder correspond one by one to the hand joints. In addition, the baseline applies the same loss function as our method.

### 4.5 Ablation study

In this section, we conduct thorough ablation experiments to assess the performance of HandGCNFormer on the Hands2017 dataset.

#### 4.5.1 HandGCNFormer modules

Table 1 presents the results of experiments conducted to assess the impact of our proposed modules, namely the Graphformer decoder and Topology-aware head. Our baseline model achieves a mean error of 7.35 mm on the “AVG” test item, representing the mean 3D distance error across all test frames. This performance is only marginally inferior to PHG, indicating that the Transformer framework effectively captures long-range context information for hand pose estimation. Subsequently, replacing the baseline decoder with our Graphformer decoder yields promising results. Leveraging the synergy between NoffGConv and the self-attention mechanism, the model equipped with the Graphformer decoder reduces the mean joint error by 0.41 mm and demonstrates a 5.4% improvement in accuracy for unseen subjects' hands. Furthermore, integrating only the Topology-aware head into the baseline model leads to significant performance gains, underscoring the importance of spatial structure perception for accurate and robust pose regression. Notably, our head achieves impressive performance without increasing the model parameters. Finally, combining our decoder and regression head in HandGCNFormer yields the best performance with the smallest model size. Particularly, HandGCNFormer surpasses the baseline by 0.69 mm in terms of mean joint error for unseen subjects' hands, highlighting its advantages in generalization.

**Table 1:** Ablation study for the effectiveness of different modules in HandGCNFormer.

**Method**	**AVG**	**SEEN**	**UNSEEN**	**Params (flops)**
Baseline	7.35	5.09	9.24	37.37M (5.81G)
+ Graphformer Decoder	6.94	4.77	8.74	33.18M (5.72G)
+ Topology-aware Head	6.90	4.67	8.77	37.24M (5.80G)
HandGCNFormer (+ both)	**6.80**	**4.64**	**8.59**	**33.04M (5.71G)**

#### 4.5.2 NoffGConv

We compare NoffGConv with other GCN variations, namely vanilla GCN (Welling and Kipf, 2016), ChebGConv (Defferrard et al., 2016), and SemGConv (Zhao et al., 2019). Table 2 presents the comparison results, where *K* denotes the order of the convolution kernel in ChebGConv. Our method outperforms other methods, highlighting the effectiveness of our NoffGConv when combined with self-attention. Furthermore, we investigate three different connection orders among the three components in the Graphformer decoder and report the results in Table 3. Here, N, S, and C represent NoffGConv, self-attention, and cross-attention, respectively. “N-S-C” corresponds to the structure of our decoder depicted in Figure 2A. “S-N-C” indicates NoffGConv is placed in the middle, while “S-C-N” signifies NoffGConv follows cross-attention. The experimental findings indicate that “N-S-C” is the optimal order for integrating NoffGConv and attention modules.

**Table 2:** Ablation study for the effectiveness of different GCN mothods in Graphformer decoder.

**Method**	**AVG**	**SEEN**	**UNSEEN**
Vanilla GCN (Welling and Kipf, 2016)	6.95	4.83	8.73
ChebGConv (*K* = 1; Defferrard et al., 2016)	6.96	4.77	8.87
ChebGConv (*K* = 2; Defferrard et al., 2016)	6.94	4.83	8.69
SemGConv (Zhao et al., 2019)	6.93	4.78	8.72
NoffGConv (ours)	**6.80**	**4.64**	**8.59**

K represents the order of convolution kernel in ChebGConv.

**Table 3:** Ablation study for the effectiveness of different connection orders between three components in the Graphformer decoder.

**Method**	**AVG**	**SEEN**	**UNSEEN**
N-S-C	**6.80**	**4.64**	**8.59**
S-N-C	6.86	4.67	8.69
S-C-N	6.86	4.68	8.68

N, S, and C denote NoffGConv, self-attention, and cross-attention, respectively. Bold values represent the best results.

### 4.6 Comparison with the state-of-the-art

#### 4.6.1 Results of 3D hand pose estimation

**Figure 4:**
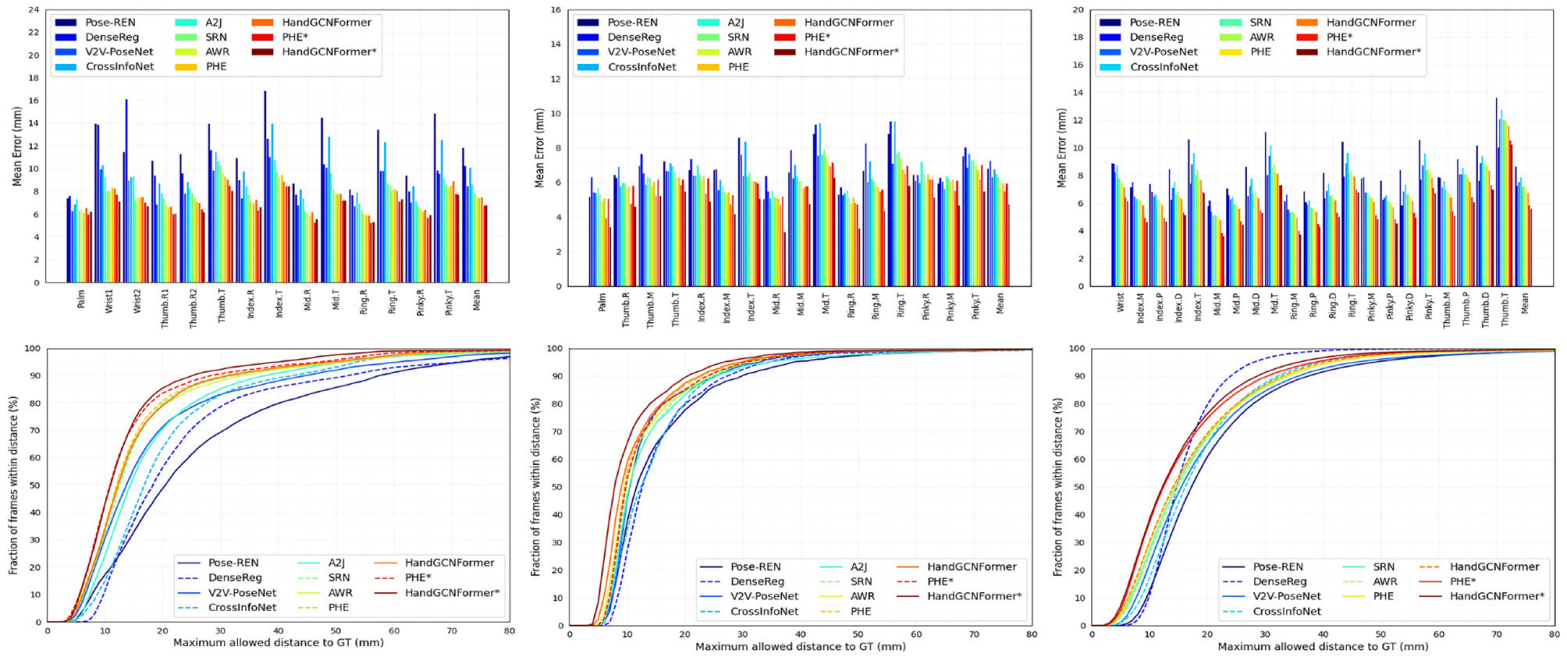
Comparison of our framework with the state-of-the-art works on NYU **(left column)**, ICVL **(middle column)**, and MSRA **(right column)** datasets. **(Top)** The mean of 3D distance error for each joint. **(Bottom)** The percentage of successful frames over different thresholds.

We compare HandGCNFormer with various existing methods (Ge et al., 2018a,b; Moon et al., 2018; Wan et al., 2018; Du et al., 2019; Ren et al., 2019, 2021a; Xiong et al., 2019; Chen et al., 2020; Huang et al., 2020c) across standard NYU, ICVL, MSRA, and Hands2017 benchmarks. Table 4 presents the comparison results using the mean 3D distance error metric. For a fair comparison, the results of previous works can be categorized into two groups. The top group results utilize the center coordinates provided by V2V-PoseNet as the hand region center for image cropping. The bottom group reports results using the average of the ground truth joints as the hand region center, denoted by “*.” Figure 4 illustrates the per-joint mean error and the percentage of successful frames across different thresholds on the NYU, ICVL, and MSRA datasets. The experimental findings demonstrate that HandGCNFormer achieves comparable or superior performance compared to other methods while maintaining real-time speed on a single GPU at 72.8 FPS. It is worth noting that the number of parameters in our model is reduced by 7.6% compared to PHG, which has 35.71 M parameters.

**Table 4:** Comparisons with state-of-the-art methods on NYU, ICVL, MSRA, and Hands2017 using the mean of 3D distance error in millimeter.

**Method**	**NYU**	**ICVL**	**MSRA**	**Hands2017**			**FPS**
				**AVG**	**SEEN**	**UNSEEN**	
DenseReg (Wan et al., 2018)	10.21	7.24	7.23^*^	-	-	-	27.8
Pose-REN (Chen et al., 2020)	11.81	6.79	8.65	-	-	-	-
HandPointNet (Ge et al., 2018a)	10.54	6.94	8.51	-	-	-	48
Point-to-Point (Ge et al., 2018b)	9.05	6.33	7.71	-	-	-	41.8
V2V-PoseNet (Moon et al., 2018)	8.41	6.28	7.59	9.95	6.97	12.43	3.5
CrossInfoNet (Du et al., 2019)	10.08	6.73	7.86	9.68	7.30	11.67	124.5
A2J (Xiong et al., 2019)	8.61	6.46	-	8.57	6.92	9.95	105.6
SRN (Ren et al., 2019)	7.79	6.27	7.17	8.39	6.06	10.33	**263.1**
AWR (Huang et al., 2020c)	7.48	5.98	7.20	7.48	5.21	9.36	-
PHG (Ren et al., 2021a)	**7.39**	5.97	6.94	7.14	5.06	8.87	58.8
HandGCNFormer	7.43	**5.48**	**6.73**	**6.80**	**4.64**	**8.59**	72.8
PHG^*^ (Ren et al., 2021a)	6.75	5.94	5.82	-	-	-	58.8
HandGCNFormer^*^	**6.74**	**4.72**	**5.57**	**5.53**	**3.74**	**7.02**	72.8

The “*.” represents that the method adopts the average of the ground truth joints as hand region center for cropping images. “SEEN” and “UNSEEN” indicate the cases whether the test subjects are involved in training set. “AVG” denotes the mean of 3D distance error over all test frames. Best in **bold**.

Specifically, on **Hands2017 dataset**, our method outperforms other methods with the mean joint error of 6.80 mm. For unseen subjects hand, our method achieves the minimum mean joint error of 8.59 mm, essentially demonstrating the excellent generalization ability of our method. In addition, HandGCNFormer^*^ improves 1.27 mm compared with HandGCNFormer in the “AVG” test case, reflecting the fact that the accuracy of hand region center coordinates limits the performance of model. On **NYU dataset**, the results of our method are comparable to PHG. This is mainly because the annotations of the NYU dataset are noisy, which limits the performance of our method in terms of all-joint mean error. Even though, our method still obtains the best performance in terms of the percentage of successful frames as shown in lower left of Figure 4. On **ICVL dataset**, HandGCNFormer and HandGCNFormer^*^ outperform the previous best results by a margin of 8.2 and 20.5%. In fact, HandGCNFormer achieves better accuracy than PHG^*^. For the per-joint error and the percentage of successful frames, our method significantly surpasses other methods under all the joints and thresholds. On **MSRA dataset**, our method is superior to PHG and PHG^*^ by a margin of 3.0 and 4.3%, respectively. Our method reduces the per-joint error and achieves the optimal percentage of successful frames under 15 mm threshold. Overall, HandGCNFormer is inherently superior to state-of-the-art methods, with a suitable trade-off between effectiveness and efficiency.

#### 4.6.2 Results of 3D hand mesh estimation

Table 5 shows that our HandGCNFormer-Mesh achieves competitive results on **HO-3D dataset**, which contain diverse hand-object occlusions. The results show that our method achieves the best results in all indicators except SC-AUC, surpassing the 3D skeleton prediction performance and 3D mesh reconstruction performance of the current state-of-the-art methods. Specifically, for SC-MPJPE and PA-MPJPE, our method leads the Keypoint Transformer method by a significant advantage of 2 and 8%, respectively, which directly proves that our proposed HandGCNFormer-Mesh has an excellent ability to predict the 3D coordinate representation of hand joints. On top of that, HandGCNFormer-Mesh has more advantages in the PA-AUC but is slightly worse than the Keypoint Transformer in the SC-AUC, and we believe that the reason is that Keypoint Transformer estimates the object pose and gesture pose at the same time. Under the constraints of the object pose, the number of successfully predicted joints accounts for a larger proportion under smaller thresholds. In addition, regarding MPVPE, our method surpasses the best mesh estimation method (Hampali et al., 2020) with an advantage of 8%, indicating the effectiveness of our proposed Topology-aware head and Graphformer Decoder structure in performing 3D hand mesh estimation tasks. On top of that, we also provide visualize results in Section 4.7.

**Table 5:** Comparison with state-of-the-art methods on HO-3D.

**Method**	**SC-MPJPE (cm)**	**PA-MPJPE (cm)**	**SC-AUC**	**PA-AUC**	**MPVPE (cm)**	***F*-score @5 mm**	***F*-score @15 mm**
I2L-MeshNet (Moon and Lee, 2020)	2.60	1.12	0.529	0.775	1.39	0.409	0.932
Pose2Mesh (Choi et al., 2020)	3.33	1.25	0.480	0.754	1.27	0.441	0.909
Hampali et al. (2020)	3.04	1.07	0.494	0.788	1.06	0.506	0.942
Hasson et al. (2019)	3.69	1.14	0.369	0.773	1.14	0.428	0.932
ArtiBoost (Li et al., 2021b)	2.53	1.14	0.532	0.773	1.09	0.488	0.944
METRO (Lin et al., 2021)	2.89	1.04	0.504	0.792	1.11	0.484	0.946
Keypoint Transformer (Hampali et al., 2022)	2.57	1.08	**0.553**	0.786	-	-	-
HandGCNFormer-Mesh (ours)	**2.52**	**0.99**	0.533	**0.802**	**0.98**	**0.523**	**0.950**

Bold values represent the best results.

### 4.7 Visualization

**Figure 5:**
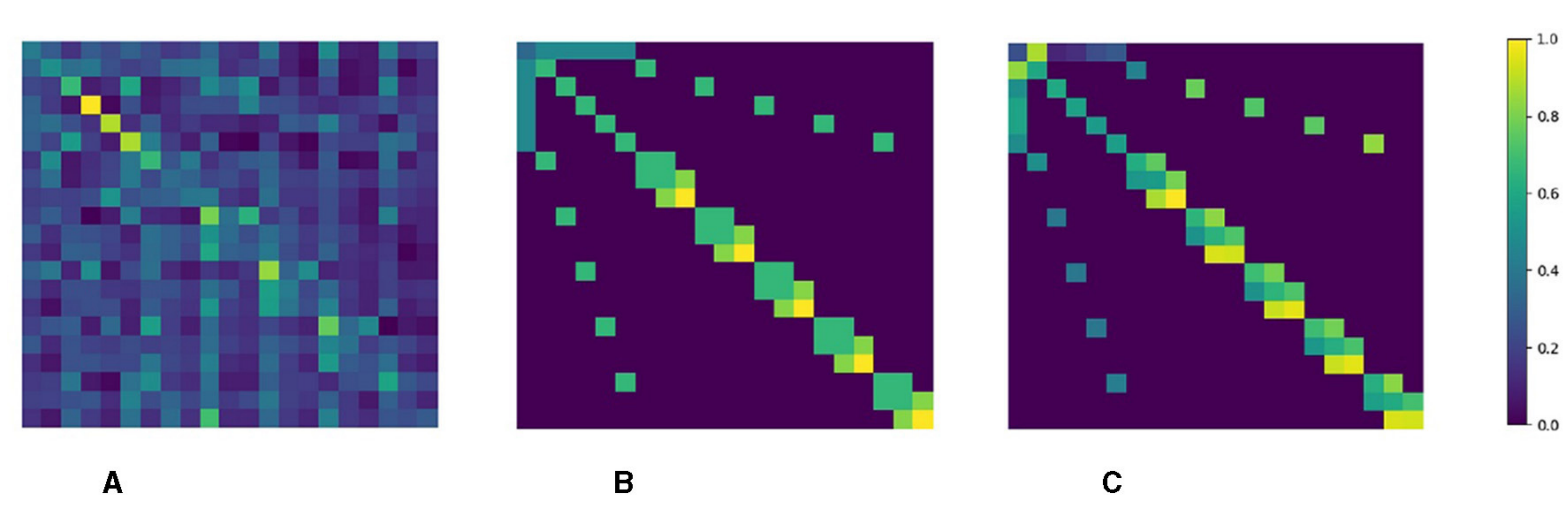
**(A)** The attention map of self-attention in decoder, dynamically models the global dependencies of joints. **(B)** Normalized adjacency matrix of NofGConv, focus on local topology perception with fixed connection strength between joints. **(C)** Learned weight matrix of SemGConv, adaptively models complex dependencies among neighboring joints.

We visualize the weight matrices of self-attention in the decoder, NoffGConv, and SemGConv during information aggregation. As depicted in Figure 5, the self-attention mechanism dynamically captures long-range dependencies between joints but overlooks the inherent topology information of the hand. On the other hand, NoffGConv and SemGConv focus on the local connection relations of hand kinematic topology. While self-attention learns the degree of dependencies between joints dynamically and flexibly, our NoffGConv assigns fixed attention to neighboring joints through a normalized adjacency matrix. In contrast, SemGConv utilizes a learned weight matrix to adaptively extract complex relationships among neighboring joints, thereby providing richer spatial constraints for pose regression.

**Figure 6:**
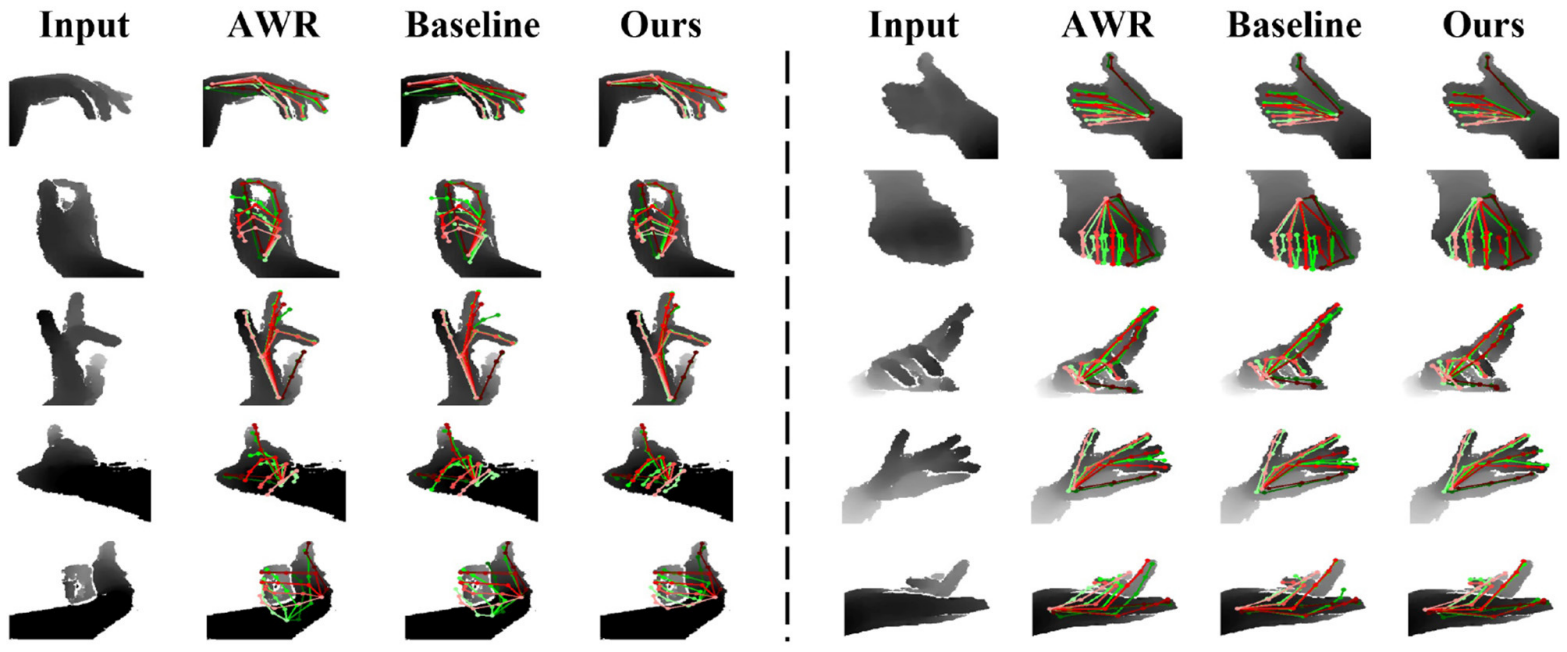
Qualitative comparison among AWR, our baseline, and our HandGCNFormer on Hands2017 dataset. **(Left)** Qualitative results of images with self-occlusion. **(Right)** Qualitative results of images with self-similarity. Red pose represents the ground truth. Green pose is predicted result.

Figure 6 presents qualitative results of samples exhibiting self-occlusion and self-similarity from the Hands2017 dataset. To ensure a fair comparison, the results of AWR are reported at the same input size and hand region center as our method. It is evident that HandGCNFormer achieves more accurate and plausible poses compared to both AWR (Huang et al., 2020c) and our strong baseline. In particular, AWR fails in extreme self-occlusion cases, while HandGCNFormer successfully identifies joint locations and produces more plausible poses guided by a global understanding of input data and prior knowledge of hand topology.

**Figure 7:**
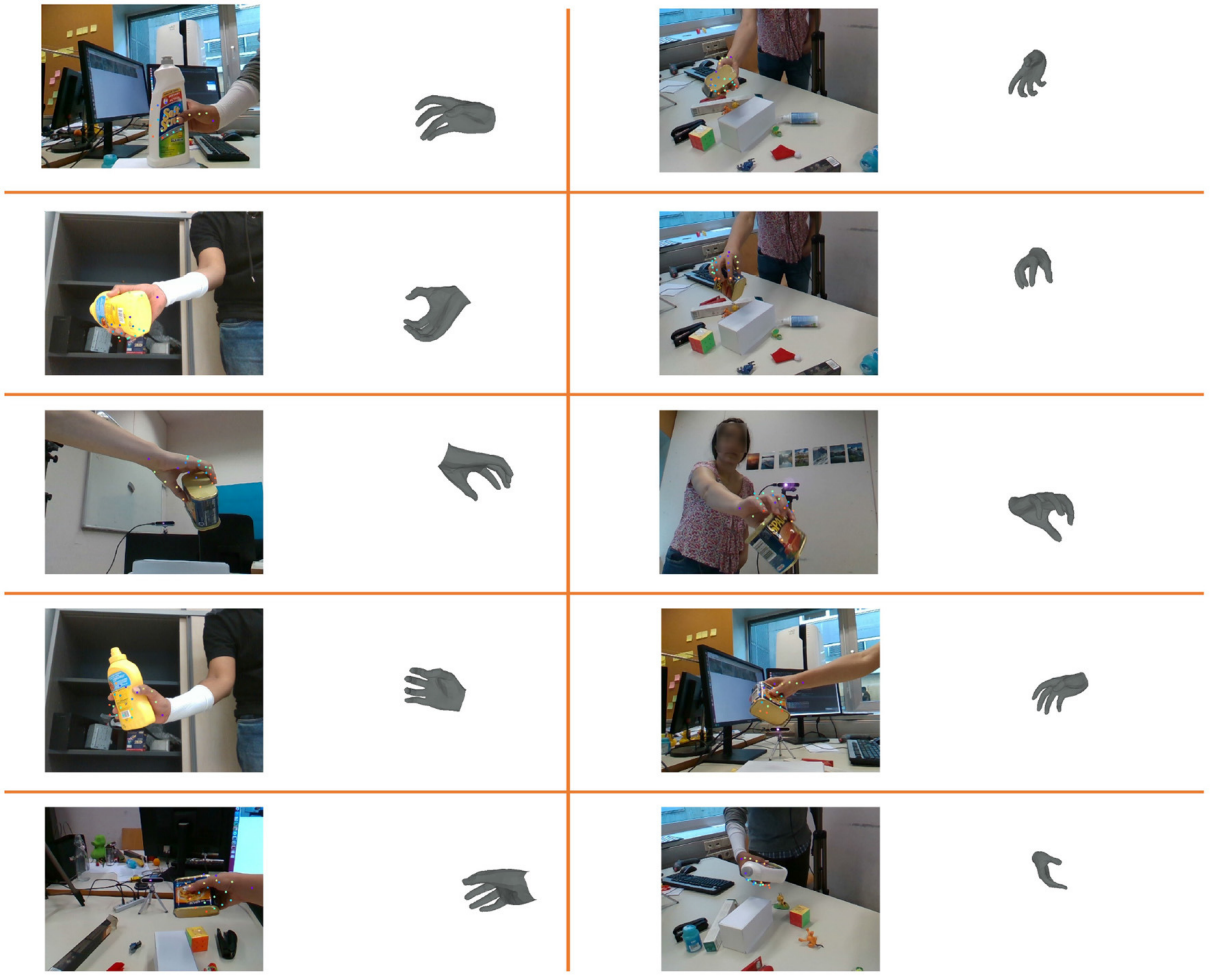
Visualization of the results on HO-3D. As the results show, our HandGCNFormer-Mesh can also complete 3D hand mesh estimation well in severe self-occlusion and self-similarity situations.

Figure 7 depicts the visualization of results on the HO-3D dataset. Even in challenging occlusion scenarios, our method consistently generates meshes that conform to the human hand topology. Furthermore, in cases of self-occlusion and self-similarity, the mesh results for individual fingers remain accurate. These observations indicate that HandGCNFormer-Mesh yields excellent results on the HO-3D dataset.

### 4.8 Comparative analysis in challenging scenarios

In the domain of 3D hand pose and mesh estimation, scenarios characterized by severe occlusions, close interactions with objects, and rapid motions present substantial challenges. These conditions often obscure critical visual information and disrupt the continuity of observable features, making accurate pose and mesh reconstruction particularly difficult. In this section, we present a comparative analysis of our proposed models, HandGCNFormer and HandGCNFormer-Mesh, against state-of-the-art methods, specifically focusing on their performance in these demanding scenarios.

#### 4.8.1 Severe occlusions

Occlusions significantly impact the visibility of hand joints, a crucial aspect for accurate pose estimation. In our evaluation on the HO-3D dataset, which includes diverse hand-object interaction scenes, HandGCNFormer-Mesh demonstrated robust performance, achieving a competitive edge over methods like Keypoint Transformer (Hampali et al., 2022). This can be attributed to the model's effective integration of kinematic topology, which aids in inferring occluded joints' positions based on the visible parts of the hand. However, in extreme cases where the majority of the hand is occluded, our model faced difficulties, suggesting room for improvement in leveraging contextual and prior shape information.

#### 4.8.2 Complex hand-object interactions

Scenarios involving intricate hand-object interactions pose challenges due to the close proximity of hand joints to objects, often leading to confusion between the object and hand features. HandGCNFormer-Mesh's topology-aware approach enables it to better distinguish between hand and object features, outperforming baseline models by inserting Shape Regressor. This success underscores the importance of incorporating structural and relational priors into the model. Future improvements could explore more sophisticated mechanisms for differentiating hand and object features, possibly through enhanced attention mechanisms or deeper integration of object recognition pathways.

#### 4.8.3 Static images implying rapid motions

While our method primarily focuses on static images, rapid hand motions can result in motion blur, which introduces additional challenges for accurate joint localization and pose estimation. Although HandGCNFormer is designed without temporal data from video sequences, its architecture demonstrates resilience against motion blur by leveraging global and local context effectively. Enhancements in handling blur through improved image processing techniques or by simulating motion effects during training could further strengthen the model's performance in these scenarios.

#### 4.8.4 Comparison with state-of-the-art methods

When comparing HandGCNFormer and HandGCNFormer-Mesh to current leading methods, our models' topology-aware nature particularly stands out in handling severe occlusions and intricate hand-object interactions, surpassing those based solely on visual feature extraction. However, as motion blur presents a distinct challenge in the absence of temporal data, there is an opportunity to explore innovations in image preprocessing or training methodologies to mitigate its impact.

#### 4.8.5 Conclusion

The comparative analysis highlights HandGCNFormer and HandGCNFormer-Mesh's effective handling of challenging scenarios through a novel topology-aware framework. While the models excel in scenarios with occlusions and hand-object interactions, addressing the effects of motion blur in static images presents an avenue for future improvements. Continued advancements in these areas will contribute to the models' robustness and accuracy in 3D hand pose and mesh estimation across a wider array of challenging conditions.

## 5 Conclusion and future research directions

This paper introduces a new Topology-aware Transformer network named HandGCNFormer, designed to accurately infer plausible 3D hand poses, particularly in scenarios involving self-occlusion and self-similarity. Within HandGCNFormer, we devised a Graphformer decoder and a Topology-aware head to optimize the collaboration between Transformer and GCN components. HandGCNFormer thoroughly captures both the global context of images and joints, along with the inherent kinematic topology of the hand, effectively mitigating uncertainties arising from obscured or closely resembling joints. Extensive experiments validate that HandGCNFormer attains state-of-the-art performance across four publicly available datasets, notably diminishing prediction errors, particularly in intricate scenarios. Furthermore, we proposed HandGCNFormer-Mesh for the 3D hand mesh estimation task on HO-3D, and the results further highlight the effectiveness and scalability of our proposed Topology-aware head and Graphformer Decoder structures on the task of 3D hand mesh estimation under different modality input.

The advancements made with HandGCNFormer and HandGCNFormer-Mesh pave the way for several promising avenues of research. While our current models show significant improvements in 3D hand pose and mesh estimation from static images, exploring the following directions could further enhance their utility and applicability:

- Improved handling of motion blur: although our work primarily addresses static images, motion blur—a common artifact in images of moving hands—presents an opportunity for future research. Developing techniques that can infer motion direction and intensity from static images to predict more accurate poses under motion blur conditions could be highly beneficial.
- Integration of semantic context: our models could benefit from incorporating broader scene context and semantic information, enabling them to better understand hand interactions within complex environments. Research into combining our topology-aware approach with semantic segmentation and object recognition could lead to more nuanced and accurate hand pose and mesh estimations.
- Cross-modal learning: exploring cross-modal learning strategies to leverage complementary information from depth, RGB, and possibly infrared data could enhance the models' robustness to varying lighting conditions and backgrounds. This approach could also help in better distinguishing between hands and objects in closely interacting scenarios.

By pursuing these directions, we aim to build upon the foundation laid by HandGCNFormer and HandGCNFormer-Mesh, pushing the boundaries of what is currently achievable in the field of 3D hand pose and mesh estimation.

## Data availability statement

The original contributions presented in the study are included in the article/supplementary material, further inquiries can be directed to the corresponding author.

## Author contributions

SY: Writing—original draft, Writing—review & editing. YW: Writing—original draft, Writing—review & editing. LC: Writing— original draft, Writing—review & editing. XZ: Supervision, Writing—review & editing. JL: Supervision, Writing—review & editing.

## References

[B1] Bai R. Li M. Meng B. Li F. Ren J. Jiang M. . (2021). GCST: graph convolutional skeleton transformer for action recognition. arXiv preprint arXiv:2109.02860. 10.48550/arXiv.2109.02860

[B2] Caramalau R. Bhattarai B. Kim T.-K. (2021). “Active learning for bayesian 3d hand pose estimation,” in Proceedings of the IEEE/CVF Winter Conference on Applications of Computer Vision, 3419–3428.

[B3] Carion N. Massa F. Synnaeve G. Usunier N. Kirillov A. Zagoruyko S. (2020). “End-to-end object detection with transformers,” in *European Conference on Computer Vision* (Berlin: Springer), 213–229.

[B4] Chen X. Wang G. Guo H. Zhang C. (2020). Pose guided structured region ensemble network for cascaded hand pose estimation. Neurocomputing 395, 138–149. 10.1016/j.neucom.2018.06.097

[B5] Choi H. Moon G. Lee K. M. (2020). “Pose2Mesh: graph convolutional network for 3d human pose and mesh recovery from a 2d human pose,” in *Computer Vision–ECCV 2020: 16th European Conference, Glasgow, UK, August 23–28, 2020, Proceedings, Part VII 16* (Berlin: Springer), 769–787.

[B6] Defferrard M. Bresson X. Vandergheynst P. (2016). Convolutional neural networks on graphs with fast localized spectral filtering. Adv. Neural Inform. Process. Syst. 29:9375. 10.48550/arXiv.1606.09375

[B7] Doosti B. Naha S. Mirbagheri M. Crandall D. J. (2020). “Hope-net: a graph-based model for hand-object pose estimation,” in Proceedings of the IEEE/CVF Conference on Computer Vision and Pattern Recognition, 6608–6617.

[B8] Dosovitskiy A. Beyer L. Kolesnikov A. Weissenborn D. Zhai X. Unterthiner T. . (2020). An image is worth 16x16 words: transformers for image recognition at scale. arXiv preprint arXiv:2010.11929. 10.48550/arXiv.2010.11929

[B9] Du K. Lin X. Sun Y. Ma X. (2019). “CrossInfoNet: multi-task information sharing based hand pose estimation,” in Proceedings of the IEEE/CVF Conference on Computer Vision and Pattern Recognition, 9896–9905.

[B10] Fang L. Liu X. Liu L. Xu H. Kang W. (2020). “JGR-P2O: joint graph reasoning based pixel-to-offset prediction network for 3d hand pose estimation from a single depth image,” in *European Conference on Computer Vision* (Berlin: Springer), 120–137.

[B11] Garcia-Hernando G. Yuan S. Baek S. Kim T.-K. (2018). “First-person hand action benchmark with RGB-D videos and 3d hand pose annotations,” in Proceedings of the IEEE Conference on Computer Vision and Pattern Recognition, 409–419.

[B12] Ge L. Cai Y. Weng J. Yuan J. (2018a). “Hand PointNet: 3d hand pose estimation using point sets,” in *2018 IEEE/CVF Conference on Computer Vision and Pattern Recognition* (Salt Lake City, UT: IEEE), 8417–8426.

[B13] Ge L. Ren Z. Yuan J. (2018b). “Point-to-point regression pointnet for 3d hand pose estimation,” in Proceedings of the European Conference on Computer Vision (ECCV), 475–491.

[B14] Glorot X. Bengio Y. (2010). “Understanding the difficulty of training deep feedforward neural networks,” in Proceedings of the Thirteenth International Conference on Artificial Intelligence and Statistics; JMLR Workshop and Conference Proceedings, 249–256.

[B15] Guo H. Wang G. Chen X. Zhang C. (2017a). Towards good practices for deep 3d hand pose estimation. arXiv preprint arXiv:1707.07248. 10.48550/arXiv.1707.07248

[B16] Guo H. Wang G. Chen X. Zhang C. Qiao F. Yang H. (2017b). “Region ensemble network: improving convolutional network for hand pose estimation,” in *2017 IEEE International Conference on Image Processing (ICIP)* (Beijing: IEEE), 4512–4516.

[B17] Hampali S. Rad M. Oberweger M. Lepetit V. (2020). “Honnotate: a method for 3d annotation of hand and object poses,” in Proceedings of the IEEE/CVF Conference on Computer Vision and Pattern Recognition, 3196–3206.22374536

[B18] Hampali S. Sarkar S. D. Rad M. Lepetit V. (2022). “Keypoint transformer: aolving joint identification in challenging hands and object interactions for accurate 3d pose estimation,” in Proceedings of the IEEE/CVF Conference on Computer Vision and Pattern Recognition, 11090–11100.

[B19] Hasson Y. Tekin B. Bogo F. Laptev I. Pollefeys M. Schmid C. (2020). “Leveraging photometric consistency over time for sparsely supervised hand-object reconstruction,” in Proceedings of the IEEE/CVF Conference on Computer Vision and Pattern Recognition, 571–580.

[B20] Hasson Y. Varol G. Tzionas D. Kalevatykh I. Black M. J. Laptev I. . (2019). “Learning joint reconstruction of hands and manipulated objects,” in Proceedings of the IEEE/CVF Conference on Computer Vision and Pattern Recognition, 11807–11816.

[B21] He K. Zhang X. Ren S. Sun J. (2016). “Deep residual learning for image recognition,” in Proceedings of the IEEE Conference on Computer Vision and Pattern Recognition, 770–778.

[B22] Huang L. Tan J. Liu J. Yuan J. (2020a). “Hand-transformer: non-autoregressive structured modeling for 3d hand pose estimation,” in *European Conference on Computer Vision* (Berlin: Springer), 17–33.

[B23] Huang L. Tan J. Meng J. Liu J. Yuan J. (2020b). “Hot-net: non-autoregressive transformer for 3d hand-object pose estimation,” in Proceedings of the 28th ACM International Conference on Multimedia, 3136–3145.

[B24] Huang W. Ren P. Wang J. Qi Q. Sun H. (2020c). “AWR: Adaptive weighting regression for 3d hand pose estimation,” in Proceedings of the AAAI Conference on Artificial Intelligence, vol. 34, 11061–11068.

[B25] Kong D. Ma H. Xie X. (2020). SIA-GCN: a spatial information aware graph neural network with 2d convolutions for hand pose estimation. arXiv preprint arXiv:2009.12473. 10.48550/arXiv.2009.12473

[B26] Li K. Wang S. Zhang X. Xu Y. Xu W. Tu Z. (2021a). “Pose recognition with cascade transformers,” in Proceedings of the IEEE/CVF Conference on Computer Vision and Pattern Recognition, 1944–1953.

[B27] Li K. Yang L. Zhan X. Lv J. Xu W. Li J. . (2021b). ArtiBoost: boosting articulated 3d hand-object pose estimation via online exploration and synthesis. arXiv preprint arXiv:2109.05488. 10.48550/arXiv.2109.05488

[B28] Li W. Liu H. Tang H. Wang P. Van Gool L. (2022). “MHFormer: multi-hypothesis transformer for 3d human pose estimation,” in Proceedings of the IEEE/CVF Conference on Computer Vision and Pattern Recognition, 13147–13156.

[B29] Li X. Wang H. Yi L. Guibas L. J. Abbott A. L. Song S. (2020). “Category-level articulated object pose estimation,” in Proceedings of the IEEE/CVF Conference on Computer Vision and Pattern Recognition, 3706–3715.34986097

[B30] Lin K. Wang L. Liu Z. (2021). “End-to-end human pose and mesh reconstruction with transformers,” in Proceedings of the IEEE/CVF Conference on Computer Vision and Pattern Recognition, 1954–1963.

[B31] Liu Z. Lin Y. Cao Y. Hu H. Wei Y. Zhang Z. . (2021). “Swin transformer: hierarchical vision transformer using shifted windows,” in Proceedings of the IEEE/CVF International Conference on Computer Vision, 10012–10022.

[B32] Loshchilov I. Hutter F. (2017). Decoupled weight decay regularization. arXiv preprint arXiv:1711.05101. 10.48550/arXiv.1711.0510138536692

[B33] Madadi M. Escalera S. Baró X. Gonzàlez J. (2022). End-to-end global to local convolutional neural network learning for hand pose recovery in depth data. IET Comput. Vis. 16, 50–66. 10.1049/cvi2.12064

[B34] Malik J. Abdelaziz I. Elhayek A. Shimada S. Ali S. A. Golyanik V. . (2020). HandVoxNet: deep voxel-based network for 3d hand shape and pose estimation from a single depth map. in Proceedings of the IEEE/CVF Conference on Computer Vision and Pattern Recognition, 7113–7122.

[B35] Mao W. Ge Y. Shen C. Tian Z. Wang X. Wang Z. (2021). TFpose: direct human pose estimation with transformers. arXiv preprint arXiv:2103.15320. 10.48550/arXiv.2103.15320

[B36] Martínez-González A. Villamizar M. Odobez J.-M. (2021). “Pose transformers (potr): human motion prediction with non-autoregressive transformers,” in Proceedings of the IEEE/CVF International Conference on Computer Vision, 2276–2284.

[B37] Moon G. Chang J. Y. Lee K. M. (2018). “V2V-PoseNet: voxel-to-voxel prediction network for accurate 3d hand and human pose estimation from a single depth map,” in Proceedings of the IEEE Conference on Computer Vision and Pattern Recognition, 5079–5088.

[B38] Moon G. Chang J. Y. Suh Y. Lee K. M. (2017). Holistic planimetric prediction to local volumetric prediction for 3d human pose estimation. arXiv preprint arXiv:1706.04758. 10.48550/arXiv.1706.04758

[B39] Moon G. Lee K. M. (2020). “I2L-MeshNet: image-to-lixel prediction network for accurate 3d human pose and mesh estimation from a single RGB image,” in *Computer Vision–ECCV 2020: 16th European Conference, Glasgow, UK, August 23–28, 2020, Proceedings, Part VII 16* (Berlin: Springer), 752–768.

[B40] Oberweger M. Lepetit V. (2017). “DeepPrior++: improving fast and accurate 3d hand pose estimation,” in Proceedings of the IEEE International Conference on Computer Vision Workshops, 585–594.29993927

[B41] Oberweger M. Wohlhart P. Lepetit V. (2015). Hands deep in deep learning for hand pose estimation. arXiv preprint arXiv:1502.06807. 10.48550/arXiv.1502.06807

[B42] Qiu L. Zhang X. Li Y. Li G. Wu X. Xiong Z. . (2020). “Peeking into occluded joints: a novel framework for crowd pose estimation,” in *European Conference on Computer Vision* (Berlin: Springer), 488–504.

[B43] Ren P. Sun H. Hao J. Qi Q. Wang J. Liao J. (2021a). Pose-guided hierarchical graph reasoning for 3-d hand pose estimation from a single depth image. IEEE Trans. Cybernet. 53, 315–328. 10.1109/TCYB.2021.3083637 34383658

[B44] Ren P. Sun H. Huang W. Hao J. Cheng D. Qi Q. . (2021b). Spatial-aware stacked regression network for real-time 3d hand pose estimation. Neurocomputing 437, 42–57. 10.1016/j.neucom.2021.01.045

[B45] Ren P. Sun H. Qi Q. Wang J. Huang W. (2019). SRN: Stacked regression network for real-time 3d hand pose estimation. BMVC 112. Available online at: https://dblp.org/rec/conf/bmvc/RenSQWH19.html

[B46] Romero J. Tzionas D. Black M. J. (2022). Embodied hands: modeling and capturing hands and bodies together. arXiv preprint arXiv:2201.02610. 10.48550/arXiv.2201.02610

[B47] Sun X. Wei Y. Liang S. Tang X. Sun J. (2015). “Cascaded hand pose regression,” in Proceedings of the IEEE Conference on Computer Vision and Pattern Recognition, 824–832.

[B48] Sun X. Xiao B. Wei F. Liang S. Wei Y. (2018). “Integral human pose regression,” in Proceedings of the European Conference on Computer Vision (ECCV), 529–545.

[B49] Tang D. Jin Chang H. Tejani A. Kim T.-K. (2014). “Latent regression forest: structured estimation of 3d articulated hand posture,” in Proceedings of the IEEE Conference on Computer Vision and Pattern Recognition, 3786–3793.

[B50] Tompson J. Stein M. Lecun Y. Perlin K. (2014). Real-time continuous pose recovery of human hands using convolutional networks. ACM Trans. Graph. 33, 1–10. 10.1145/2629500

[B51] Tunga A. Nuthalapati S. V. Wachs J. (2021). “Pose-based sign language recognition using GCN and BERT,” in Proceedings of the IEEE/CVF Winter Conference on Applications of Computer Vision, 31–40.

[B52] Vaswani A. Shazeer N. Parmar N. Uszkoreit J. Jones L. Gomez A. N. . (2017). Attention is all you need. Adv. Neural Inform. Process. Syst. 30:3762. 10.48550/arXiv.1706.03762

[B53] Wan C. Probst T. Van Gool L. Yao A. (2018). “Dense 3d regression for hand pose estimation,” in Proceedings of the IEEE Conference on Computer Vision and Pattern Recognition, 5147–5156.

[B54] Wang Y. Chen L. Li J. Zhang X. (2023). “HandGCNFormer: a novel topology-aware transformer network for 3d hand pose estimation,” in Proceedings of the IEEE/CVF Winter Conference on Applications of Computer Vision, 5675–5684.

[B55] Welling M. Kipf T. N. (2016). “Semi-supervised classification with graph convolutional networks,” in *J. International Conference on Learning Representations (ICLR 2017)*.

[B56] Xiong F. Zhang B. Xiao Y. Cao Z. Yu T. Zhou J. T. . (2019). “A2J: Anchor-to-joint regression network for 3d articulated pose estimation from a single depth image,” in Proceedings of the IEEE/CVF International Conference on Computer Vision, 793–802.

[B57] Yan S. Xiong Y. Lin D. (2018). “Spatial temporal graph convolutional networks for skeleton-based action recognition,” in *Thirty-Second AAAI Conference on Artificial Intelligence*.

[B58] Yang S. Quan Z. Nie M. Yang W. (2020). Transpose: towards explainable human pose estimation by transformer. arXiv preprint arXiv:2012.14214 2. 10.48550/arXiv.2012.14214

[B59] Yuan S. Ye Q. Garcia-Hernando G. Kim T.-K. (2017). The 2017 hands in the million challenge on 3d hand pose estimation. arXiv preprint arXiv:1707.02237. 10.48550/arXiv.1707.02237

[B60] Zhao L. Peng X. Tian Y. Kapadia M. Metaxas D. N. (2019). “Semantic graph convolutional networks for 3d human pose regression,” in Proceedings of the IEEE/CVF Conference on Computer Vision and Pattern Recognition, 3425–3435.

[B61] Zhu X. Su W. Lu L. Li B. Wang X. Dai J. (2020). Deformable DETR: deformable transformers for end-to-end object detection. arXiv preprint arXiv:2010.04159. 10.48550/arXiv.2010.04159

